# Delays in accessing high-quality care for newborns in East Africa: An analysis of survey data in Malawi, Mozambique, and Tanzania

**DOI:** 10.7189/jogh.14.04022

**Published:** 2024-02-09

**Authors:** Lori Niehaus, Ashley Sheffel, Henry D Kalter, Agbessi Amouzou, Alain K Koffi, Melinda K Munos

**Affiliations:** Johns Hopkins Bloomberg School of Public Health, Department of International Health, Baltimore, Maryland, USA

## Abstract

**Background:**

Despite the existence of evidence-based interventions, substantial progress in reducing neonatal mortality is lagging, indicating that small and sick newborns (SSNs) are likely not receiving the care they require to survive and thrive. The ‘three delays model’ provides a framework for understanding the challenges in accessing care for SSNs. However, the extent to which each delay impacts access to care for SSNs is not well understood. To fill this evidence gap, we explored the impact of each of the three delays on access to care for SSNs in Malawi, Mozambique, and Tanzania.

**Methods:**

Secondary analyses of data from three different surveys served as the foundation of this study. To understand the impact of delays in the decision to seek care (delay 1) and the ability to reach an appropriate point of care (delay 2), we investigated time trends in place of birth disaggregated by facility type. We also explored care-seeking behaviours for newborns who died. To understand the impact of delays in accessing high-quality care after reaching a facility (delay 3), we measured facility readiness to manage care for SSNs. We used this measure to adjust institutional delivery coverage for SSN care readiness.

**Results:**

Coverage of institutional deliveries was substantially lower after adjusting for facility readiness to manage SSN care, with decreases of 30 percentage points (pp) in Malawi, 14 pp in Mozambique, and 24 pp in Tanzania. While trends suggest more SSNs are born in facilities, substantial gaps remain in facilities’ capacities to provide lifesaving interventions. In addition, exploration of care-seeking pathways revealed that a substantial proportion of newborn deaths occurred outside of health facilities, indicating barriers in the decision to seek care or the ability to reach an appropriate source of care may also prevent SSNs from receiving these interventions.

**Conclusions:**

Investments are needed to overcome delays in accessing high-quality care for the most vulnerable newborns, those who are born small or sick. As more mothers and newborns access health services in low- and middle-income countries, ensuring that life-saving interventions for SSNs are available at the locations where newborns are born and seek care after birth is critical.

While globally, there has been great success in increasing child survival, the decline in under-five mortality over the last three decades has not been uniform among ages. In 2020, nearly half (47%) of all under-five deaths occurred during the neonatal period (first 27 days of life), up from just 40% in 1990 [1]. In addition, deaths within the neonatal period are not evenly distributed by age, with nearly three-fourths of all newborn deaths occurring within the first week of life [1]. Despite widespread recognition that neonatal survival must be prioritised, 61 countries are not on track to meet their neonatal mortality rate target for the third sustainable development goal – no more than 12 neonatal deaths per 1000 live births by 2030 [2].

Notwithstanding these setbacks, effective interventions exist to save newborn lives. The Every Newborn Action Plan, endorsed by 194 United Nations (UN) member states and over 80 partners, identified two evidence-based packages of care with the highest impact for ending preventable newborn deaths – labour, delivery and first week of life care, and small and sick newborn care [3]. Small and sick newborns (SSNs) – those weighing less than 2500 g at birth (including preterm and small for gestational age newborns) or having any medical or surgical condition [4] – are at the highest risk of mortality [5–7]. However, several effective interventions for the care of SSNs have been associated with significant reductions in neonatal mortality risk [8-12]. These life-saving interventions largely require facility-based care [8,9], and thus, SSNs must be able to access timely, high-quality care at health facilities.

Despite the existence of evidence-based interventions, substantial progress in reducing neonatal mortality has not been made, indicating that SSNs are likely not receiving the care they require to survive and thrive when and where they need it. The Three Delays Model provides a valuable framework for understanding the challenges in accessing care for SSNs. The model was first proposed by Thaddeus and Maine in 1994 to understand the proximal causes of maternal mortality and access to care [13]. It breaks down barriers in the care-seeking process into three categories: delays in the decision to seek care, delays in reaching an appropriate source of care (usually a health facility), and delays in receipt of adequate and appropriate care after reaching the facility. Understanding the relative impact of these delays on newborns' access to health services can help guide context-specific policies and interventions.

The extent to which these delays impact access to care for SSNs is not well understood, and there is minimal information available on care-seeking behaviours for newborns. Previous studies have explored increases in institutional delivery coverage [14–17] that suggest more newborns are reaching health facilities during the critical window of labour, delivery, and the first week of life, thus overcoming delays one and two immediately after birth. However, few studies include detailed information on the types of facilities, including level (e.g. hospitals or primary facilities) and managing authority (e.g. government or private), from which care is sought. A more granular understanding of these facility types and more information on care-seeking behaviours for newborns after birth would identify delays in reaching the appropriate level of care and allow for more targeted intervention strategies.

Additionally, understanding only where newborns seek care is insufficient to assess whether they could access necessary, high-quality services after reaching the appropriate facility (i.e. whether they experienced delay three). Many factors may influence the receipt of adequate care for SSNs in health facilities, including inadequate staffing, poor availability of equipment, and low health worker competency to deliver SSN care interventions [18]. Input-adjusted and quality-adjusted coverage measures can be used to provide evidence to assess whether newborns who reached health facilities may still face delays in receipt of high-quality services, due to poor newborn health service availability or service provision [19,20]. Poor facility readiness – the availability of inputs at a health facility that are required to deliver newborn health services – may result in delays to SSNs receiving the care they need. Still, we are unaware of any studies that have explicitly adjusted coverage for facility readiness to provide lifesaving interventions for SSNs.

To fill these evidence gaps, we analysed available survey and social autopsy data from Malawi, Mozambique, and Tanzania to better understand the impact of the three delays on access to high-quality care for SSNs. We investigated time trends in place of birth disaggregated by facility type (level and managing authority) and explored care-seeking behaviours for newborns who died to understand the impact of delays in the decision to seek care (delay 1) and the ability to reach an appropriate point of care (delay 2). We measured overall and intervention-specific readiness to manage care for SSNs disaggregated by facility type and used the resulting scores to adjust institutional delivery coverage for SSN care readiness to understand the potential of newborn service input availability to lead to delays in SSN care after reaching a facility (delay 3). Together, with these analyses we aimed to explore where interventions targeting neonatal survival may be most impactful.

## METHODS

### Country selection

We selected three countries in East Africa based on their neonatal mortality burdens and the availability of verbal and social autopsy data (Malawi, Mozambique, and Tanzania). All three have neonatal mortality rates above the global average of 17 deaths per 1000 live births (19 per 1000 in Malawi, 28 per 1000 in Mozambique, and 20 per 1000 in Tanzania) and thus far have not demonstrated sufficient progress to meet the Sustainable Development Goal neonatal mortality rate target for 2030 (Target 3.2) [1].

### Data sources

Secondary analyses of data from three types of surveys served as the foundation of this study: 1) household surveys (HH), 2) health facility assessments (HFA), and 3) verbal and social autopsy interviews (VASA) (**Table 1**).

**Table 1:** Data sources

Survey type	Source	Used in analysis
Household survey		
*Malawi*	DHS 2000, DHS 2004, MICS 2006, DHS 2010, MICS 2013–14, DHS 2015–16	Time trends of the place of birth, service contact and readiness-adjusted institutional delivery coverage (DHS 2015–16)
*Mozambique*	DHS 2003, DHS 2011, AIS 2015	Time trends of the place of birth, service contact and readiness-adjusted institutional delivery coverage (AIS 2015)
*Tanzania*	DHS 2004–05, DHS 2010, AIS/MIS 2011–12, DHS 2015–16	Time trends of the place of birth, service contact and readiness-adjusted institutional delivery coverage (DHS 2015–16)
Health facility assessment		
*Malawi*	SPA 2013–14	Facility readiness scores, service contact and readiness-adjusted institutional delivery coverage
*Mozambique*	SARA 2018	Facility readiness scores, service contact and readiness-adjusted institutional delivery coverage
*Tanzania*	SPA 2014–15	Facility readiness scores, service contact and readiness-adjusted institutional delivery coverage
Verbal and social autopsy interview		
*Malawi*	CHERG* Stillbirth, Neonatal, and Child VASA questionnaire, 2013	Care-seeking behaviours for newborns who died
*Mozambique*	COMSA† Stillbirth, Neonatal, Child and Adult VASA questionnaire, 2018–20	Care-seeking behaviours for newborns who died
*Tanzania*	CHERG Stillbirth, Neonatal, and Child VASA questionnaire, 2017–18	Care-seeking behaviours for newborns who died

AIS – AIDS Indicator Survey, CHERG – Child Health Epidemiology and Reference Group, COMSA – Countrywide Mortality Surveillance for Action, DHS – Demographic and Health Survey, MICS – Multiple Indicator Cluster Survey, MIS – Malaria Indicator Survey, SARA – Service Availability and Readiness Assessment, SPA – Service Provision Assessment

*The Child Health Epidemiology and Reference Group (CHERG) was a technical advisory group that worked with international health institutions to improve ‘evidence on the causes and determinants of maternal, neonatal, and child morbidity and mortality, on intervention coverage, and on the effectiveness of interventions to inform and influence global priorities and programmes [21].

†Countrywide Mortality Surveillance for Action (COMSA) is a national sample registration system for mortality and causes of death monitoring implemented by through a collaboration between the Mozambique National Institute of Health and the Institute for International Programs at Johns Hopkins Bloomberg School of Public Health. Data collection has been ongoing since roll-out began in March 2018 [22].

HH data and questionnaires, specifically Demographic and Health Surveys (DHS), AIDS Indicator Surveys, and Multiple Indicator Cluster Surveys (MICS) are publicly available through the DHS Program (DHS and AIDS Indicator Surveys) [23] or from the United Nations Children’s Fund (UNICEF) (Multiple Indicator Cluster Surveys) [24]. HFA data access varied by country, with Service Provision Assessments (SPA) used for Malawi and Tanzania publicly available through the DHS Program [23], and the Mozambique 2018 Service Availability and Readiness Assessment (SARA) accessed with permission from the Mozambique World Health Organization (WHO) Country Office and the Mozambique National Institute of Health. We accessed VASA de-identified data and questionnaires with permission from the primary research team at Johns Hopkins University.

All included household surveys were nationally representative. The Malawi 2013–14 SPA [25] and the Mozambique 2018 SARA [26] were censuses of all formal-sector health facilities in each country. The Tanzania 2014–15 SPA [27] was a sample of facilities stratified by region and facility type (level and managing authority). VASA data for Mozambique and Tanzania were nationally representative, whereas data for Malawi was collected in two districts (Balaka and Salima) located in the South and Central regions of Malawi and was representative of those two districts (Table S1 in the **Online Supplementary Document**). Additional information on sampling design and data collection procedures is contained within the survey reports for HHs and HFAs [25–40] and in publications by the primary research teams for VASA [22,41,42].

### Defining the Three Delays to newborn care

We adapted the original framework on the Three Delays to define the delays in access to care for newborns after birth. We followed the same approach used in previous studies focused on this population (**Table 2**) [43–45].

**Table 2:** Definitions of delays in access to newborn care

Delay 1*
Use service contact coverage of institutional delivery calculated from household surveys to understand the proportion of newborns who, at the time of and immediately after delivery, do not face delays 1 and 2 because they have already reached the facility at the time of birth.
**Delay 2†**
Use newborn VASA data to understand the frequency with which caretakers of deceased newborns born at home or at a health facility and then discharged after birth, sought care for their newborn after birth and reported concerns or barriers to care-seeking. In the analysis, we could not clearly distinguish between newborns who died and faced delay 1 vs delay 2.
**Delay 3‡**
Use readiness-adjusted coverage of institutional delivery to measure if required inputs for newborn services are available in health facilities where newborns are born. We did not have the data to measure newborn receipt of appropriate, high-quality care (quality-adjusted coverage). However, readiness-adjusted coverage remains important as without critical inputs in health facilities, newborns are likely to face delay 3.

VASA – verbal and social autopsy survey

*A delay in the caretaker’s decision to seek care for their newborn from a qualified health provider/facility.

†A delay in the newborn physically reaching a qualified health provider/facility.

‡A delay in the ability of a newborn to receive high-quality and appropriate health services after reaching a health provider/facility.

### Data analysis

#### National time trends in place of birth

In each country, we mapped place of birth response options in HH questionnaires over time to generate a set of health facility categories for which it was possible to generate time trends for place of birth. Across analyses, we defined facility type by the facility level (e.g. secondary/hospital, primary/health centre, health post) and managing authority (e.g. government, private, religious). However, a few household surveys collected less detailed information on facility level and managing authority, leading to broader facility-type categories in those settings.

We first calculated from each survey the service contact coverage of institutional delivery defined as the proportion of the most recent live births of interviewed women in the two, three, or five years preceding the survey that took place in a health facility of any type. The Malawi MICS 2006 and Malawi MICS 2013-14 only collected information on births within the two years preceding the surveys. The Mozambique AIS 2015 only collected information on births within 32 months preceding the survey. Next, for each survey year, we disaggregated institutional deliveries by level alone (primary vs secondary) and by more detailed health facility type categories described above. All analyses accounted for the HH survey design (clustering, stratification, and survey weights).

#### Care-seeking behaviours for newborns who died

**Figure 1:**
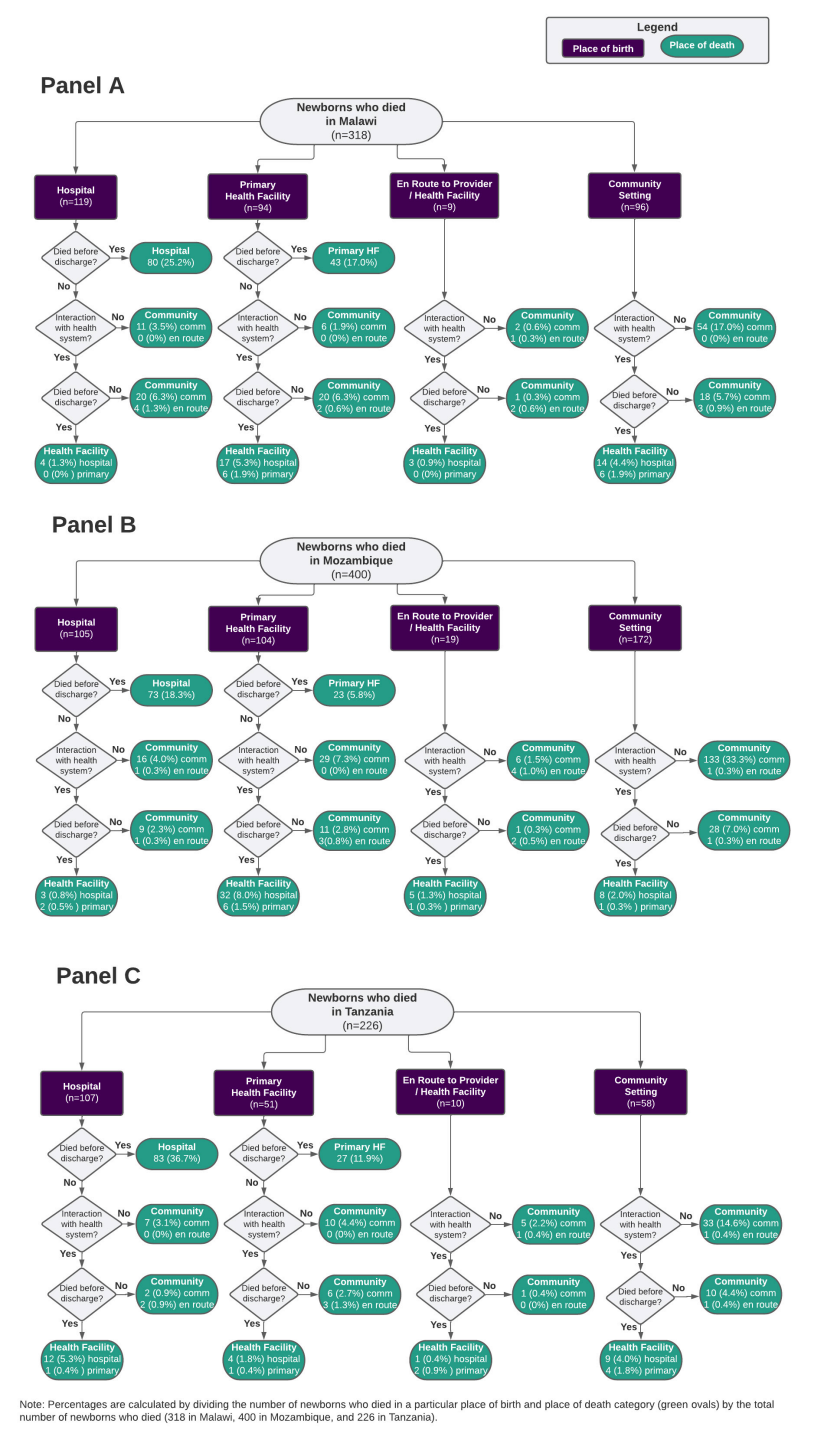
Care-seeking itinerary for newborns’ fatal illnesses from birth to death. **Panel A**. Malawi. **Panel B**. Mozambique. **Panel C**. Tanzania.

We used VASA data to understand the care-seeking itinerary for newborns who died (**Figure 1**). We first disaggregated newborns by their place of birth (hospital, primary health facility, or community (including en route births)). Then, we calculated the proportion of newborns who were born in a health facility and died before discharge from the facility in which they were born. Among newborns born in facilities who were discharged before death and newborns born in the community, we determined the proportion who sought care from a health facility, respectively, after discharge or birth, and of these newborns, the proportions who ultimately died at a hospital, primary health facility, or in the community. All newborns of caretakers who sought care after a home birth or discharge from the health facility where they gave birth were treated equally, regardless of the number of facilities from which they sought care. We calculated the proportion of newborns born in the community who never interacted with the health system (i.e. never sought facility-based care for any reason). For caretakers of newborns who died, we calculated the proportion who reported having concerns related to delays 1 and 2 in the Three Delays Model that affected their decision and ability to seek care for their newborn’s fatal illness.

#### Facility readiness to manage small and sick newborn care

We used the work of Moxon et al. [18,46,47], WHO guidance [8], and selected HFA questionnaires [25–27] to develop a measure of facility readiness to manage care for SSNs. Due to limited data availability, we were unable to include all SSN care interventions identified in the literature in this measure. Ultimately, we included seven interventions required to care for SSNs: essential newborn care (a package of interventions expected to be provided to every newborn at the time of delivery and includes drying, hygienic cord care, vitamin K, and eye care), breastfeeding, resuscitation, prevention of mother-to-child transmission of HIV, kangaroo mother care, antibiotics for infection, and a cross-cutting domain for general readiness items (Table S3 in the **Online Supplementary Document**). Because most of these items were collected in the labour and delivery module of the HFA questionnaire, we limited our analysis to facilities offering labour, delivery and newborn services (55% of facilities in Malawi, 85% in Mozambique, 80% in Tanzania) (Tables S1 in the **Online Supplementary Document**).

For each facility included in the analysis, we defined binary (0 and 1) indicators for each item, indicating whether the item was available and functional/non-expired at the time of the HFA. Binary indicators were created for all readiness items except those derived from health provider sections of the HFAs. These indicators are defined as the proportion of health providers providing delivery or newborn care services that have been trained in or carried out a particular intervention within a specified period. For each HFA, we calculated intervention-specific readiness scores for each of the six intervention domains and the cross-cutting domain as an unweighted average of available items in that domain. We then calculated an overall SSN care readiness score for each HFA by averaging the domain scores. We disaggregated overall SSN readiness scores by health facility type. All analyses accounted for the HFA survey design (stratification and survey weights), as applicable.

#### Readiness-adjusted coverage of institutional deliveries for SSN care

To calculate readiness-adjusted institutional delivery coverage for small and sick newborn care in each country, we linked data on place of birth from the most recent HH survey (2015 for all countries) to the overall and intervention-specific SSN care readiness scores calculated from HFA data. HFAs for this analysis were selected based on the time period of HFA data collection overlapping with the reference period for the place of birth question in the country’s HH survey. For Mozambique, because no HFA met this criterion, the 2018 SARA was used.

We used an ecological approach, as validated in previous studies [48–50], to link each birth in the HH data sets to stratum-specific intervention-specific and overall readiness scores calculated from HFAs. First, we mapped response options for place of birth in the HH survey onto health facility types from the HFAs for each country. We then assigned each birth in the HH data set to a stratum, where the stratum was defined as the place of birth (health facility type or home) and administrative area (a district in Malawi, a province in Mozambique, a region in Tanzania). Based on the assigned stratum, each birth was linked to a SSN care readiness score, calculated as the stratum average readiness score from the corresponding HFA. For example, a baby born at a public health centre in the Mwanza region, Tanzania, would have been assigned the average readiness score for all public health centres in the Mwanza region included in the Tanzania 2014–15 SPA. For 30% (51/168) of administrative area-facility type pairs for Malawi, 27% (9/33) for Mozambique, and 42% (151/360) for Tanzania, the national average readiness score for all facilities of a given type was used because there was no data collected in the HFA for any facility of the given type in the particular administrative area. Women who did not deliver in health facilities were assigned a readiness score of 0.

To estimate readiness-adjusted coverage of institutional deliveries in each country, we assigned each birth a value of 0 or 1 (0 if the birth took place at home and 1 if the birth took place at a health facility of any type). We multiplied this value by the readiness score (both overall and intervention-specific) for each birth. At a population level, this approach reduces the institutional birth coverage by the gap between measured and perfect facility readiness and accounts for differences in facility readiness by facility type. We calculated readiness-adjusted coverage at the national level and by facility type by taking the mean of the products (readiness score multiplied by zero or one), accounting for both HH and HFA survey designs using the HH weights to generate point estimates and a jackknife approach to estimate the standard errors, where the standard error was derived from the distribution generated by withholding each HH cluster and each health facility [51]. We also disaggregated this analysis by whether the baby died in the neonatal period; however, as we found no statistically significant differences between babies who died in the newborn period (days 0–27) and those who survived to day 28, we present these analyses for all live births.

#### Statistical software

All analyses were completed using Stata/SE, version 17.0 (Stata Corp LLC, College Station, TX, USA) [52], *R*, version 4.0.3 (R Core Team, Vienna, Austria) [53], and R Studio (R Studio Team, Boston, MA, USA) [54].

## RESULTS

### National time trends in place of birth

**Figure 2:**
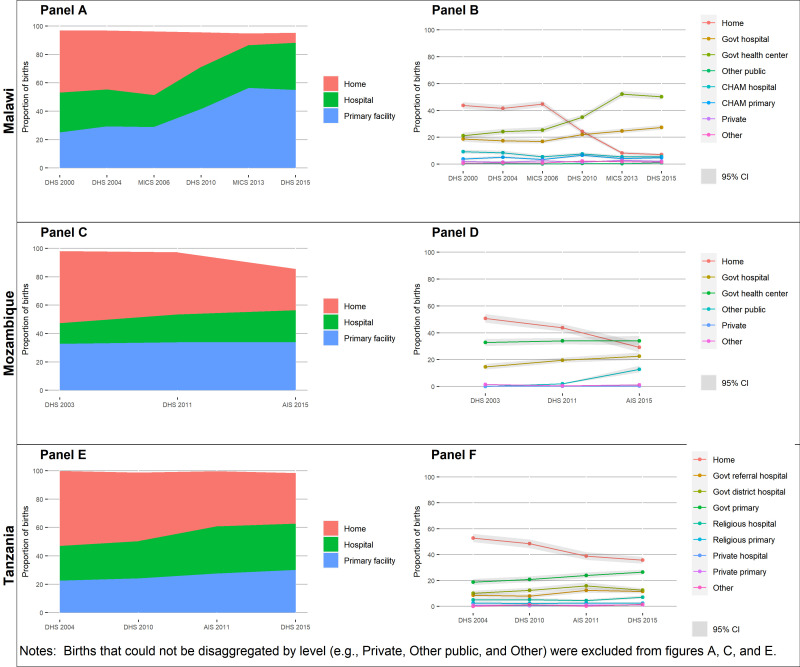
Proportion of births by place of birth, over time. **Panes A–B**. Malawi. **Panels C–D**. Mozambique. **Panels E–F**. Tanzania.

Across all three countries, the proportion of babies born at home substantially decreased over time as more births shifted to health facilities (**Figure 2** and Table S2 in the **Online Supplementary Document**). The largest decrease in the proportion of births occurring at home was in Malawi, which experienced a 37% decrease in home births between 2000 and 2015, followed by Mozambique (22%) and Tanzania (17%) **(****Figure 2**, Panels A, C, E). In Malawi, this decrease occurred rapidly from 2006–13 before levelling off, with just 7% of births occurring at home by 2015. In Mozambique and Tanzania, comparatively, the decrease over time was relatively modest, resulting in 29% of births in Mozambique and 36% of births in Tanzania occurring at home by 2015. Breaking down the distribution of births by facility level and managing authority revealed that the decline in home births from 2000 to 2015 in Malawi and Tanzania was largely related to increases in births at public (government) primary health facilities (29% increase in Malawi and 8% in Tanzania), with smaller increases at public hospitals (9% increase in Malawi and 5% in Tanzania) and virtually no change in the non-governmental and private sectors (**Figure 2**, Panels B, D, F). In Mozambique, a larger increase was seen in the proportion of births at public hospitals (5% increase) compared to public primary facilities (1% increase), although similar to Malawi and Tanzania, the largest proportion of health facility births throughout the time period remained at public primary facilities. While there was also a large increase in the proportion of births at other public health facilities in Mozambique from 2011–15, this was an artefact of the categorisation of health facilities in the 2015 AIDS Indicator Survey.

### Care-seeking behaviours for newborns who died

Exploring the care-seeking behaviours for newborns who died provides information on whether these newborns reached hospitals and/or primary health facilities around delivery, illness, and death. Across countries, at least half of the deceased newborns born in health facilities (hospital or primary) died before discharge (58% in Malawi, 46% in Mozambique, and 70% in Tanzania) (Figure S1 in the **Online Supplementary Document**). The percentage of these deceased newborns who died at their birth facility is much higher for births at hospitals compared to primary facilities (21 percentage points (pp) higher in hospitals compared to primary facilities in Malawi, 48 pp higher in Mozambique, and 25 pp higher in Tanzania). While some deceased newborns were discharged after birth and died in the community without further interaction with the health system, many returned to a health facility (or followed a referral to another facility) after they were discharged from their birth facility. Most of the newborns who were discharged from their birth facilities did not develop their fatal illnesses until after discharge (94% in Malawi, 69% in Mozambique, 83% in Tanzania). Among deceased newborns who were born outside of health facilities (i.e. in a community setting or en route to a facility), the majority in all three countries had no interaction with the formal health system before death (56% in Malawi, 77% in Mozambique, and 57% in Tanzania). Another 3–4% of newborns in each country died on route to a health facility. Among caretakers who sought care for the newborn’s fatal illness from at least one qualified provider, the median length of delay 1, or the caretaker-reported time from the newborn’s onset of symptoms to their decision to seek care for the newborn was four hours (interquartile range (IQR) = 1–24) in Malawi, zero hours (IQR = 0–24) in Mozambique, and one hour (IQR = 0.75–4) in Tanzania. The median length of delay 2, or the caretaker-reported time from the decision to seek care for their newborn’s fatal illness and seeking care for the newborn from the first health provider, was one hour (IQR = 0.33–4) in Malawi, 0.33 hours (IQR = 0–1.0) in Mozambique, and 0.5 hours (IQR = 0.08–1.5) in Tanzania.

To better understand why care was not sought for many newborns’ fatal illnesses, we analysed concerns related to delays in seeking facility-based care reported by caretakers of newborns who died. These concerns included a perceived lack of need, challenges in reaching health service delivery points, and quality of care (Figure S2 in the **Online Supplementary Document**). Notably, in all three countries, over half of all these caretakers (69% in Malawi, 59% in Mozambique, and 71% in Tanzania) did not report any concerns that deterred them from seeking care. A wide range of concerns were identified among caretakers who did express concerns. In Malawi, the most commonly reported concerns were that someone other than the caretaker was responsible for making this decision and that care-seeking took too much time away from the caretaker’s regular duties. In Tanzania, the most reported concerns included these same two concerns, in addition to concerns that the newborn was too sick to travel and would die no matter what. In Mozambique, the most cited concerns were the perception that the newborn was not sick enough to need health care and that health facilities were too far to travel. The mean number of concerns identified by caretakers with at least one concern was much higher for Malawi (x̄ = 11.96, standard deviation (SD) = 0.97) and Tanzania (x̄ = 12.29, SD = 0.85) than for Mozambique (x̄ = 1.42, SD = 0.63). Here, the differences in means may be related to differences in data collection methods between CHERG VASA (Malawi and Tanzania) and COMSA VASA (Mozambique). Data on concerns was collected by the data collector marking all applicable concerns from a checklist based on an open-ended response from the respondent.

### Facility readiness to manage small and sick newborns

**Figure 3:**
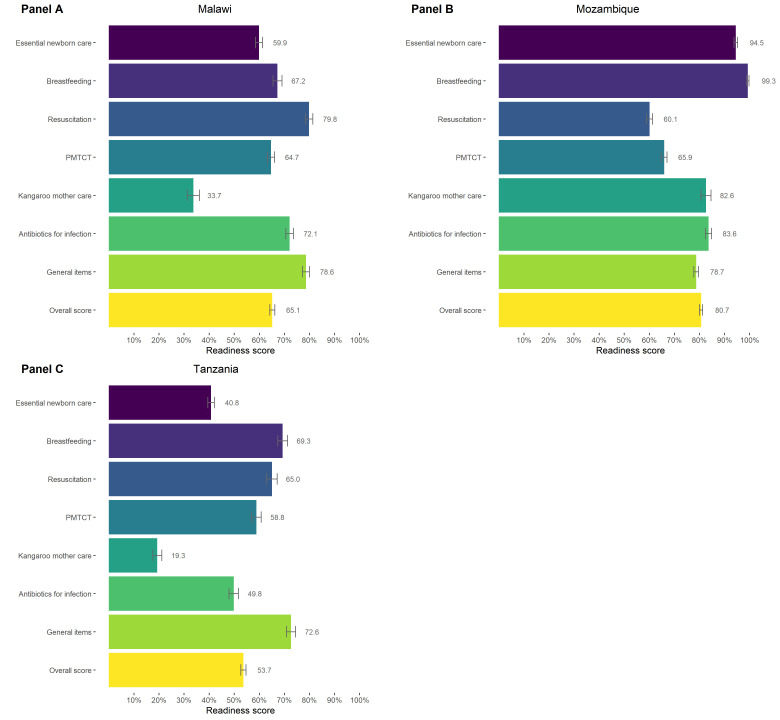
Overall and intervention-specific facility readiness scores to manage small and sick newborn care. **Panel A**. Malawi. **Panel B**. Mozambique. **Panel C**. Tanzania

Facility readiness scores provide insight into the third delay of the Three Delays Model, with lower scores indicating SSNs may face greater delays in accessing high-quality, appropriate care after reaching a health facility due to inputs required for SSN health interventions not being available at the facility. An intervention-specific readiness score of 100 would indicate that, on average, health facilities in a given country had all items measured for that intervention available at the time of the HFA (Table S3 in the **Online Supplementary Document**). No facilities in Malawi and Tanzania and only a small proportion of facilities in Mozambique (2.6%) were ready to deliver SSN care services (i.e. a readiness score of 100%). The overall SSN care facility readiness mean scores ranged from 53.7 out of 100 (95% confidence interval (CI) = 52.6–54.7) in Tanzania to 65.1 (95% CI = 64.1–66.1) in Malawi and 80.7 in Mozambique (95% CI = 80.1–81.3) (**Figure 3**). In Malawi, intervention-specific readiness was highest for resuscitation and general items (79.8 and 78.6, respectively) and lowest for kangaroo mother care (33.7). In Mozambique, breastfeeding and essential newborn care readiness scores were over 94, while resuscitation readiness was 60.1. Tanzania had the highest intervention-specific readiness score for general items (72.6), followed by breastfeeding (69.3) and resuscitation (65.0), while the lowest intervention-specific readiness score was for kangaroo mother care (19.3).

**Figure 4:**
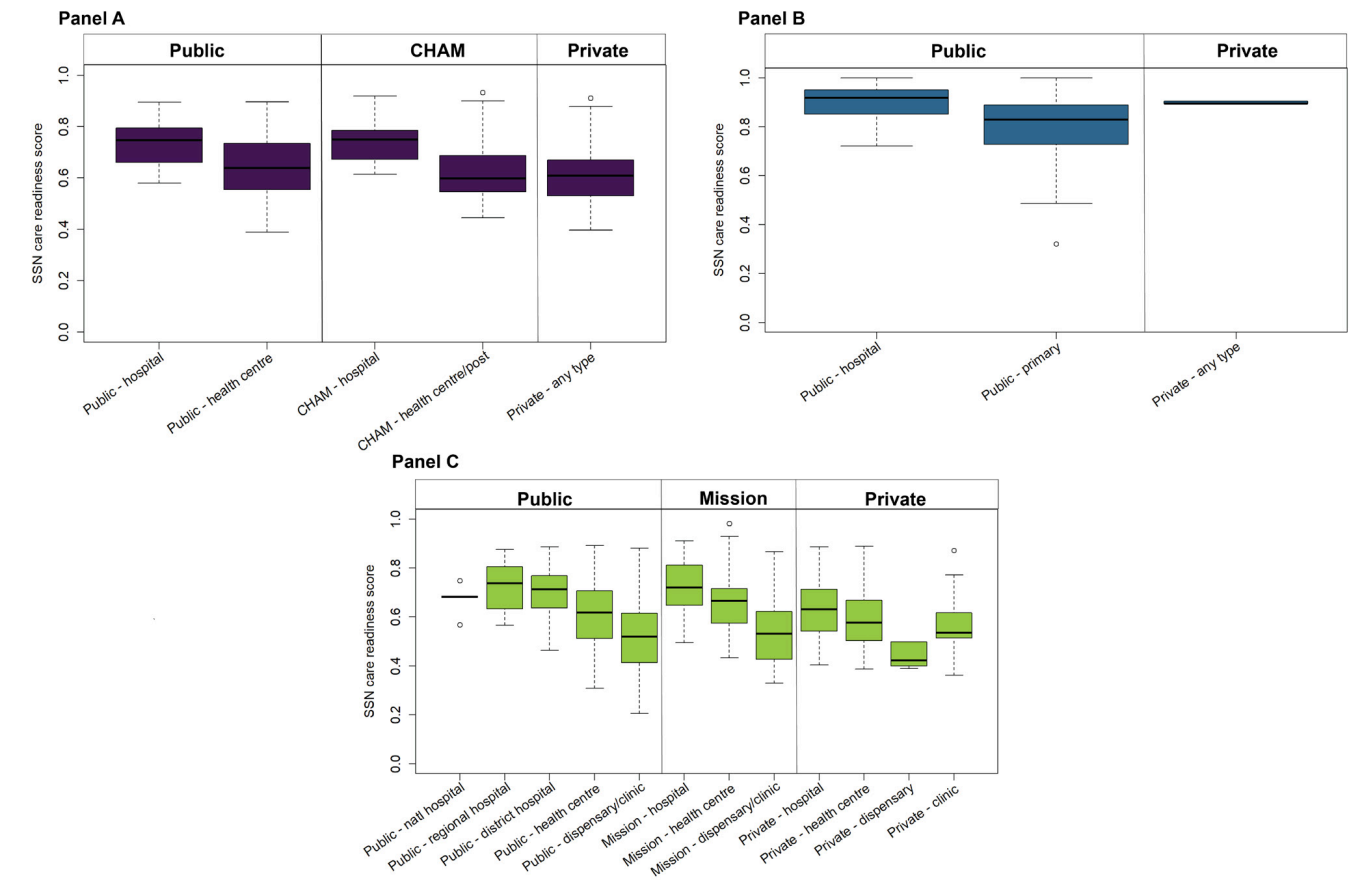
Overall readiness score to care for small and sick newborns by health facility type. **Panel A**. Malawi. **Panel B**. Mozambique. **Panel C**. Tanzania.

**Figure 5:**
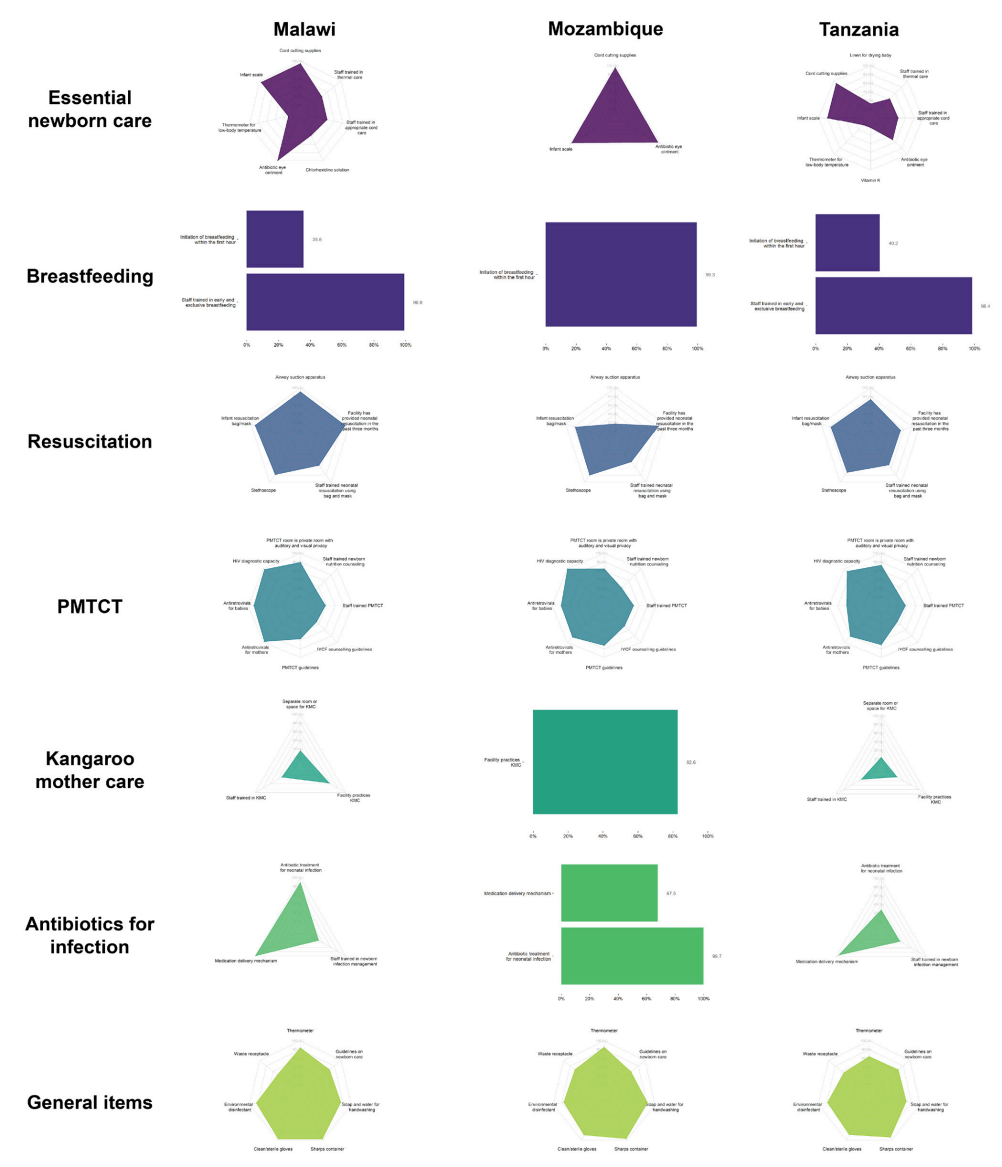
Availability of readiness items to manage small and sick newborns, by intervention, in Malawi, Mozambique, and Tanzania.

For all three countries, readiness varied by facility type, with hospitals generally more ready to care for small and sick newborns than primary health facilities (**Figure 4**). Across countries, within each managing authority category, facility readiness decreased as facility level decreased. In Malawi, the average overall facility readiness among hospitals was 74 pp, compared to 63 pp for lower-level or primary facilities. In Mozambique, the overall readiness of hospitals was 90 pp, compared to 80 pp in primary facilities, and in Tanzania, the overall readiness of hospitals was 69% compared to 55% among primary facilities. There were larger differences between referral and lower-level facilities in Tanzania, with a 21 pp gap between regional hospitals and dispensaries/clinics. The national hospitals in Tanzania had lower scores relative to regional hospitals (6 pp difference) driven by poor availability of items for kangaroo mother care. Generally, facilities of the same level but different managing authorities performed similarly, although, in Tanzania, overall SSN care readiness in private hospitals (63%) was more comparable to public primary health centres (61%) than to public referral hospitals (67%-73%). Due to survey limitations, we could not disaggregate private facilities by facility level in Malawi and Mozambique, but private facilities generally had lower readiness on average than other facility types. The wide variability in SSN care readiness in Mozambique's private facilities resulted from very few private facilities in the data set (three out of 1391 total facilities in the analysis) (**Figure 5**; Table S4 in the **Online Supplementary Document**).

In Malawi, across interventions, there was a low proportion of staff recently trained in neonatal care, including resuscitation (52%), thermal care (43%), cord care (43%), prevention of mother-to-child transmission of HIV (38%), newborn nutrition counselling (31%), kangaroo mother care (29%), and infection management (28%). This trend was not limited to Malawi, with Mozambique and Tanzania showing similar gaps in the availability of trained health workers (**Figure 5**, Panels A–C). In all three countries, there were gaps in basic commodities, although the specific items varied by country and included waste receptacles in Malawi and Tanzania and soap and water for handwashing in Mozambique. There were large gaps in the availability of items such as a thermometer for low body temperature and Infant and Young Child Feeding counselling guidelines across countries. It is important to note that there is variation in the number of items included in interventions for each country driven by the availability of data in the selected HFAs (Table S3 in the **Online Supplementary Document**). The Mozambique HFA generally included fewer items for each intervention, whereas the Tanzania HFA included some additional items for interventions for which availability was particularly low (linen for drying the baby and vitamin K).

### Readiness-adjusted coverage of institutional delivery

**Figure 6:**
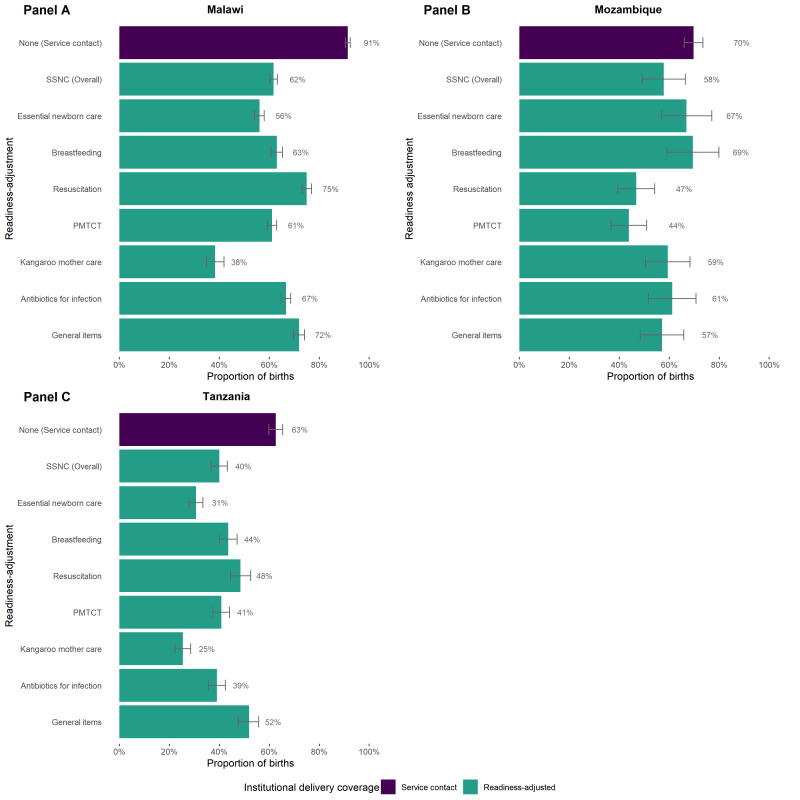
Service contact and small and sick newborn care readiness-adjusted institutional delivery coverage. **Panel A**. Malawi. **Panel B**. Mozambique. **Panel C**. Tanzania.

After adjusting for readiness to manage small and sick newborns, coverage of institutional delivery in all three countries declined (**Figure 6**). The largest gap between unadjusted and overall readiness-adjusted coverage was in Malawi, where the vast majority of births (91%, 95% CI = 90–92) took place in health facilities. After adjusting for overall facility readiness to manage SSN care, coverage dropped to 62% (95% CI = 60–63), a gap of 30 pp. In Malawi, most of the intervention-specific readiness-adjusted coverage levels were similar to the overall readiness-adjusted coverage level, with the exception of kangaroo mother care (38%, 95% CI = 35–42). For Mozambique, overall SSN care readiness-adjusted institutional delivery coverage was 56% (95% CI = 49–66), 14 pp lower than the service contact coverage value (70%, 95% CI = 66–73). Intervention-specific readiness-adjusted coverage values were relatively variable in Mozambique, ranging from 44% (95% CI = 37–51) for the prevention of mother-to-child transmission of HIV to 69% (95% CI = 59–80) for breastfeeding. For Tanzania, overall SSN care readiness-adjusted coverage was 40% (95% CI = 37–43), a gap of 24 pp compared to the service contact coverage value of 63% (95% CI = 60–65). Intervention-specific readiness-adjusted coverage was also variable in Tanzania, ranging from 25% (95% CI = 22–29) for kangaroo mother care to 52% (95% CI = 48–56) for general items.

**Figure 7:**
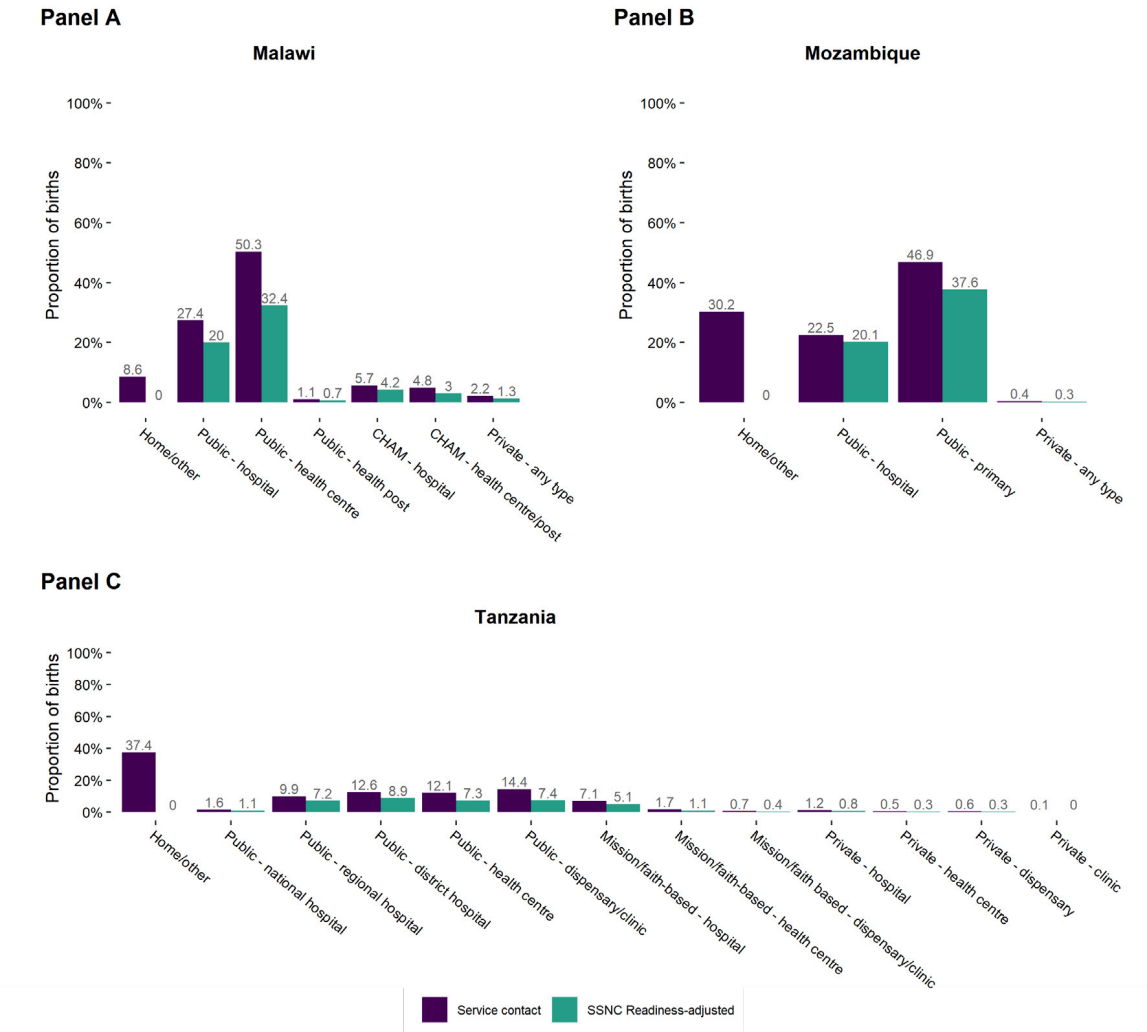
Service contact and small and sick newborn care readiness-adjusted place of birth distribution. **Panel A**. Malawi. **Panel B**. Mozambique. **Panel C**. Tanzania.

Comparing unadjusted and overall SSN care readiness-adjusted proportions of births categorised by birth location revealed gaps in effective coverage for all facility types in Malawi, Mozambique, and Tanzania (**Figure 7**). In Malawi, where most women gave birth in public primary health facilities, there was an 18 pp drop in coverage at public primary facilities and an 8 pp drop in coverage at public hospitals. In Mozambique, there was a small drop (2 pp) in coverage at hospitals but a larger drop of 9 pp at primary facilities, where most facility births occurred. In Tanzania, there were significant gaps in readiness at all levels, including nearly a 50% reduction in readiness-adjusted coverage at public dispensaries.

## DISCUSSION

This study provides evidence that small and sick newborns in Malawi, Mozambique, and Tanzania may face substantial delays in access to high-quality care. We found that institutional delivery coverage is substantially lower after adjusting for facility readiness to manage small and sick newborn care, with decreases of 30 pp in Malawi, 14 pp in Mozambique, and 24 pp in Tanzania. While delivery trends suggest more SSNs may reach facilities during the critical window of labour, delivery and the first week of life – overcoming the first and second delays in the Three Delays Model at the time of birth – these newborns may remain unable to access lifesaving interventions. Therefore, strategies aimed at addressing the delay in receipt of adequate and appropriate care after reaching a health facility (delay 3), particularly in the specific types of facilities where the largest proportion of newborns are born, would be particularly effective in these country contexts.

Our results support existing literature that show an increase in the proportion of births taking place in facilities over time in low- and middle-income countries [14,16]. Multiple studies have explored individual-level and systems-level drivers of this trend [14–16,55,56], which include deliberate efforts by governments to increase access to facility-based delivery care [16,57]. The impact of efforts to increase facility deliveries on neonatal mortality has been debated [58,59], and given low SSN care facility readiness at many facilities shown in this study, efforts to simply improve facility access at the time of birth in these countries are unlikely to be sufficient to substantially reduce neonatal mortality. While our results showed higher readiness among hospitals, many hospitals still lacked readiness items required for the effective delivery of interventions for small and sick newborns. Thus, strategies focusing only on shifting deliveries to hospitals are also likely inadequate. Broader strategies, which include quality improvement initiatives at facilities, must also be considered. These strategies should target the facilities where newborns are born, such as first-level government facilities that offer delivery services. Our analysis corroborates findings by Wang et al. [60] that the increase in facility deliveries in Malawi, Mozambique, and Tanzania is largely attributed to increases in deliveries at public first-level facilities.

Key areas for quality improvement of health facilities identified in our analysis include ensuring the availability of basic commodities for newborns and sustaining a health workforce adequately trained in neonatal interventions. Since the conception of Every Newborn Action Plan, stakeholders have identified limited health worker numbers and capacity as key barriers to the implementation of newborn care interventions [18,44,61–65]. Countries and partners have employed a variety of strategies to address this limitation [66], but recent studies show that providers in low- and middle-income countries still have relatively poor knowledge of newborn care interventions [67,68]. Variation in intervention-specific readiness scores within the three countries in our analysis suggests that different interventions may need to be prioritised for each country to maximise the impact on neonatal survival. These interventions should be considered in both public and private health facilities, as our results showed facilities of the same level, regardless of managing authority, had similar readiness to deliver SSN care, contradicting a common perception that private health facilities offer better care than public facilities. In one exception, private hospitals in Tanzania had lower readiness to deliver SSN care compared to public referral hospitals. Additionally, as large proportions of newborns in Mozambique (29%) and Tanzania (36%) continue to be born in the community, efforts to improve the quality of care at facilities will only be most effective when combined with interventions targeting the first and second delays to enable newborns to reach services at health facilities.

Our analysis of care-seeking behaviours for newborns who died in Malawi, Mozambique, and Tanzania showed that many of these newborns who were born in facilities died before discharge, likewise suggesting the facilities in which they were born may not have been adequately prepared to manage their care (delay 3). While a much larger percentage of newborns born at hospitals died at their place of birth, compared to newborns born at primary facilities, this likely reflects birth complications or other difficult conditions which may be associated with seeking out delivery care from a hospital. Other studies have explored this relationship and have shown that delays pregnant women face reaching the most capable delivery facility may contribute to the mortality of their newborns, especially if there are complications [69,70]. In this study, we centred on the newborn experience after birth, while we recognise delays in care-seeking faced by the newborn’s mother during pregnancy, labour, and delivery before birth may ultimately impact newborn health outcomes. This analysis also revealed that a substantial number of newborns who died never reached health facilities for care, and others were discharged from a health facility after birth but ultimately died at home without further care-seeking. Some of these deaths may be attributed to delays in the decision to seek care (delay 1) and the ability to access an appropriate source of care, including inadequately functioning referral systems (delay 2). Across countries, caretakers reported similar concerns around accessing health services to those identified by other studies in low- and middle-income countries contexts, such as distance to the health facility, costs associated with care-seeking, including opportunity costs from time away from regular duties, and no one available to accompany the caretaker [44,71–75]. Many respondents in Malawi and Tanzania also noted they did not have a need to seek care, as they thought traditional care was more appropriate. Other studies using social autopsy data have also reported a preference for traditional care (and the need to administer traditional and allopathic care sequentially as opposed to simultaneously) as a barrier to care-seeking [72,76]. Given that over half of all caretakers in our data sets reported no concerns related to care-seeking for their newborn’s fatal illness, influencers on decision-making around newborn care-seeking should be further explored.

While this analysis provided useful insights into access to care for small and sick newborns in Malawi, Mozambique, and Tanzania, we noted several limitations. Because this study relied on available survey data, the timeliness of survey data, lack of standardisation of surveys across time and countries, small sample sizes (VASA), and data availability (HFAs) were limitations. Limited availability of data on items required for newborn care interventions with significant variation by country HFA – which limited comparability of readiness scores between countries in our analysis – and lack of consensus on guidelines to define interventions required for SSN care have been previously documented by Moxon and colleagues [46,47]. In their analysis of the most used service availability assessment tools (including SPA and SARA), they found the neonatal interventions best represented in these tools are those promoted via vertical programming, including neonatal resuscitation, essential newborn care, and prevention of mother-to-child transmission of HIV. The focus of SPA and SARA on items available in lower-level facilities, and correspondingly our analysis on first-level interventions for SSNs expected to be available in these facilities, is likely to mask some of the readiness differential between hospitals, which provide secondary- and tertiary-level SSN care interventions, and primary facilities and may lead to an underestimate of readiness-adjusted coverage in countries with high proportions of hospital deliveries. However, given that none of the countries in this analysis had large proportions of hospital deliveries, the effect of this bias on the overall readiness-adjusted estimates would likely have been minimal. We were also not able to capture components of effective referral and transportation.

In addition, we were unable to measure the full impact each of the three delays may have on access to care for SSNs in Malawi, Mozambique, and Tanzania. Since we did not have survey data that captured our target population of SSNs, we used all births as our population of interest, which may mask care-seeking differences between SSNs and other newborns. Likewise, without data on the proportion of newborns who needed each SSN service, we used institutional delivery as our service contact coverage measure. Among newborns who were born in health facilities, discharged after birth, and died at home without further care-seeking, most became sick after discharge and likely delayed care-seeking (delay 1 or 2). However, among the newborns who were sick before discharge, it is unclear if the facility was unable to provide adequate care (delay 3) or if the illness worsened upon returning home and further care-seeking was delayed (delays 1 or 2). Likewise, for newborns who were born in health facilities, discharged, and ultimately returned to a health facility where they died, it is unknown if they delayed care-seeking (delay 1 or 2), if the facility did not provide adequate care (delay 3), or both. For the third delay, we could not measure the full effective coverage cascade as proposed by Amouzou et al. [19] and the WHO working group [20], and while readiness is a necessary input to high-quality care, it does not indicate if newborns receive high-quality services. Due to data limitations, our analysis stopped at readiness-adjusted coverage. Although this is not the final endpoint of the effective coverage cascade, it does provide valuable information on SSN care readiness, while information on intervention delivery and quality at the service delivery level remains limited in low- and middle-income countries. While our findings must be interpreted in light of these limitations, this study provides useful insights into the extent to which each of the delays of the Three Delays Model may impact access to care for SSNs in Malawi, Mozambique, and Tanzania.

## CONCLUSIONS

This analysis suggests that if we are to achieve the vision set out in Every Newborn Action Plan and meet the third sustainable development goal targets by 2030, substantial investment must be made to overcome delays in access to care for the most vulnerable newborns, those who are born small or sick. Addressing limited access to high-quality care for SSNs, who account for a disproportionate fraction of total neonatal mortality, is essential to drive down neonatal mortality rates by the end of the decade. As more women and newborns have access to health services in low- and middle-income countries, ensuring life-saving interventions for small and sick newborns are available at the locations where newborns are born and seek care after birth is critical. Future research to address data gaps in HFAs, provide additional information on care-seeking behaviours, and assess facility readiness and quality to provide SSN care in particular geographic areas would help inform the development and prioritisation of locally tailored interventions to improve neonatal survival.

## Additional material


Online Supplementary Document.
