# Supplementary material for: Delays in accessing high-quality care for newborns in East Africa: An analysis of survey data in Malawi, Mozambique, and Tanzania
Source: J Glob Health. 2024 Feb 9;14:04022. doi: 10.7189/jogh.14.04022 (PMC10854463; doi:10.7189/jogh.14.04022)
Supplement: Online Supplementary Document. [file jogh-14-04022-s001.pdf]

Online Supplementary Document

Table S1. Survey details. Surveys used for analyses, including sample sizes and representativeness. Additional details on survey design and data collection procedures for household surveys and health facility assessments can be found in corresponding survey reports.

Country	Survey	Sample size, survey	Sample size, analysis	Representativeness
**National time trends in place of birth**				
Household surveys (HH)		Interviewed women aged 15-49	Live births[2]	Geographic representativeness
Malawi	DHS 2000	13,220	11,886	National, regional, district
	DHS 2004	11,698	10,901	National, regional, selected districts
	MICS 2006	26,259	10,288	National, regional, district
	DHS 2010	23,020	19,880	National, regional, district
	MICS 2013-14	24,230	7,494	National, regional, district
	DHS 2015-16	24,562	17,148	National, regional, district
Mozambique	DHS 2003	12,418	10,294	National, regional, provincial
	DHS 2011	13,745	10,938	National, regional, provincial
	AIS 2015	6,946	2,837	National, regional, provincial
Tanzania	DHS 2004-05	10,329	8,554	National, regional
	DHS 2010	10,139	7,989	National, regional
	AIS/MIS 2011-12	10,967	5,833	National, zonal, regional
	DHS 2015-16	13,266	10,144	National, zonal, regional
**Facility readiness scores and readiness-adjusted institutional delivery coverage**				
Health facility assessments (HFA)		Health facilities	Health facilities	Geographic representativeness

		assessed	assessed offering delivery services[1]	
Malawi	SPA 2013-14	977	540	Census, all levels
Mozambique	SARA 2018	1,643	1,391	Census, all levels
Tanzania	SPA 2014-15	1,188	951	National, regional, facility type (level and managing authority)
Household surveys (HH)		Interviewed women aged 15-49	Most recent live births[2]	Geographic representativeness
Malawi	DHS 2015-16	24,562	13,399	National, regional, district
Mozambique	AIS 2015	6,946	2,648	National, regional, provincial
Tanzania	DHS 2015-16	13,266	7,025	National, zonal, regional
**Care-seeking behaviors for newborns who died**				
Verbal and social autopsy questionnaires (VASA)		Newborns who died	Newborns who died whose place of death was not 'en route'	Geographic representativeness
Malawi	CHERG Stillbirth, Neonatal, and Child VASA questionnaire, 2013	320	318	Selected districts (Balaka and Salima)
Mozambique	COMSA Stillbirth, Neonatal, Child	403	400	National

[1] HFA analytic data sets were limited to include only facilities offering delivery and newborn care services (Malawi and Tanzania) or basic obstetric care services (Mozambique).
[2] Analyses were limited to live births to interviewed women in the five years preceding the survey for which information on place of delivery was available (not missing), except for MWI MICS 2013-14 and MWI MICS 2006 which included only live births in the preceding two years and MOZ AIS 2015 which included only live births in the preceding 32 months.

	and Adult VASA questionnaire, 2018			
Tanzania	CHERG Stillbirth, Neonatal, and Child VASA questionnaire, 2017/18	228	226	National

Table S2. Proportion of births by place of birth, over time in Malawi, Mozambique, and Tanzania.

Malawi						
**Survey**	**Place of birth (Level only)**	**Estimate**	**95% CI**	**Place of birth (Level & managing authority)**	**Estimate**	**95% CI**
**2015 DHS**	home	7.1	[6.2-8.0]	home	7.1	[6.2-8.0]
	hospitals	33.1	[31.2-35.1]	govt hospital	27.4	[25.7-29.2]
				CHAM hospital	5.7	[4.6-6.9]
	primary	55.1	[53.1-57.1]	govt health center	50.2	[48.1-52.4]
				CHAM health center/ primary	4.8	[3.9-5.9]
	other	4.8	[4.1-5.5]	other public	1.1	[0.8-1.4]
				private	2.2	[1.7-2.9]
				other	1.5	[1.2-1.8]
**2013 MICS**	home	8.3	[7.2-9.5]	home	8.3	[7.2-9.5]
	hospitals	30.2	[28.2-32.3]	govt hospital	24.7	[23.0-26.5]
				cham hospital	5.5	[4.5-6.7]
	primary	56.4	[54.2-58.6]	govt health center	52.2	[49.8-54.5]
				cham health center/ primary	4.2	[3.5-5.1]
	other	5.1	[4.4-6.0]	other public	0.4	[0.2-0.8]
				private	2.9	[2.3-3.7]
				other	1.8	[1.5-2.3]
**2010 DHS**	home	24.3	[22.6-26.1]	home	24.3	[22.6-26.1]
	hospitals	29.7	[27.8-31.6]	govt hospital	22	[20.3-23.7]
				cham hospital	7.7	[6.6-8.9]
	primary	41.6	[39.4-43.7]	govt health center	34.9	[32.9-37.0]
				cham health center/ primary	6.6	[5.5-7.9]

	other	4.5	[3.9-5.1]	other public	0.6	[0.4-0.9]
				private	1.6	[1.2-2.1]
				other	2.3	[2.0-2.6]
**2006 MICS**	home	44.8	[42.6-47.1]	home	44.8	[42.6-47.1]
	hospitals	22.5	[20.8-24.3]	govt hospital	17	[15.4-18.6]
				cham hospital	5.5	[4.5-6.8]
	primary	28.9	[26.8-31.1]	govt health center	25.3	[23.0-27.7]
				cham health center/primary	3.6	[3.0-4.4]
	other	3.8	[3.1-4.5]	other public	0.3	[0.2-0.6]
				private	2.5	[2.0-3.2]
				other	0.9	[0.7-1.2]
**2004 DHS**	home	41.6	[39.2-44.0]	home	41.6	[39.2-44.0]
	hospitals	25.9	[23.4-28.5]	govt hospital	17.4	[15.5-19.5]
				cham hospital	8.5	[7.1-10.1]
	primary	29.4	[27.4-31.6]	govt health center	24.2	[22.3-26.3]
				cham health center/primary	5.2	[4.2-6.5]
	other	3.1	[2.5-3.8]	other public	0.4	[0.2-0.6]
				private	1.6	[1.2-2.3]
				other	1.1	[0.9-1.4]
**2000 DHS**	home	43.7	[41.2-46.3]	home	43.7	[41.2-46.3]
	hospitals	28.1	[25.6-30.8]	govt hospital	18.8	[16.6-21.1]
				cham hospital	9.3	[8.0-10.8]
	primary	25.1	[22.7-27.6]	govt health center	21.2	[19.0-23.6]
				cham health center/primary	3.9	[3.1-4.8]
	other	3.1	[2.4-4.0]	other public	0.4	[0.2-0.7]
				private	2	[1.4-2.9]
				other	0.8	[0.6-1.0]
**Mozambique**						
**2015 AIS**	home	29.1	[25.5-33.0]	home	29.1	[25.5-33.0]
	hospitals	22.5	[19.9-25.3]	govt hospital	22.5	[19.9-25.3]
	primary	34	[30.6-37.7]	govt health center	34	[30.6-37.7]
	other	14.4	[12.0-17.2]	other public	12.8	[10.5-15.6]
				private	0.4	[0.2-0.8]
				other (non-specified)	1.2	[0.8-1.8]
**2011 DHS**	home	43.8	[41.0-46.6]	home	43.8	[41.0-46.6]
	hospitals	19.6	[17.8-21.6]	govt hospital	19.6	[17.8-21.6]
	primary	33.9	[31.5-36.3]	govt health center	33.9	[31.5-36.3]
	other	2.7	[2.3-3.2]	other public	2	[1.7-2.4]
				private	0.2	[0.1-0.3]
				other (non-specified)	0.6	[0.4-0.8]
**2003 DHS**	home	50.7	[47.5-54.0]	home	50.7	[47.5-54.0]
	hospitals	14.6	[12.6-17.0]	govt hospital	14.6	[12.6-17.0]
	primary	32.8	[30.2-35.5]	govt health center	32.8	[30.2-35.5]

	other	1.8	[1.5-2.3]	other public	0.1	[0.0-0.5]
				private	0.2	[0.1-0.4]
				other (non-specified)	1.5	[1.2-1.8]
**Tanzania**						
**2015 DHS**	home	35.7	[33.0-38.4]	home	35.7	[33.0-38.4]
	hospitals	32.5	[30.3-34.8]	govt hospital - national/ regional referrral	11.5	[10.1-13.0]
				govt hospital - district	12.6	[11.2-14.2]
				religious - secondary	7.1	[6.0-8.5]
				private- secondary	1.2	[0.9-1.6]
	primary	30.2	[28.3-32.1]	govt - primary (health center, dispensary, clinic)	26.5	[24.7-28.4]
				religious - primary	2.5	[1.9-3.3]
				private - primary	1.2	[0.8-1.7]
	other	1.7	[1.4-2.1]	other	1.7	[1.4-2.1]
**2011 AIS**	home	38.8	[35.9-41.9]	home	38.8	[35.9-41.9]
	hospitals	33.3	[30.6-36.2]	govt hospital - national/ regional referrral	12.5	[10.6-14.7]
				govt hospital - district	15.9	[13.5-18.6]
				religious - secondary	4.5	[3.6-5.5]
				private- secondary	0.5	[0.3-0.8]
	primary	27.6	[25.3-30.1]	govt - primary (health center, dispensary, clinic)	24	[21.8-26.3]
				religious - primary	2.6	[1.9-3.5]
				private - primary	1.1	[0.8-1.5]
	other	0.2	[0.1-0.4]	other	0.2	[0.1-0.4]
**2010 DHS**	home	48.4	[45.0-51.7]	home	48.4	[45.0-51.7]
	hospitals	26.2	[23.4-29.2]	govt hospital - national /regional referrral	7.9	[6.5-9.7]
				govt hospital - district	12.4	[10.6-14.5]
				religious - secondary	5.3	[4.2-6.6]
				private- secondary	0.6	[0.2-1.9]
	primary	24.2	[21.8-26.7]	govt - primary (health center, dispensary, clinic)	20.9	[18.7-23.3]
				religious - primary	2.3	[1.6-3.3]
				private - primary	1	[0.7-1.6]
	other	1.2	[0.9-1.6]	other	1.2	[0.9-1.6]
**2004 DHS**	home	52.8	[49.6-56.1]	home	52.8	[49.6-56.1]
	hospitals	24.4	[21.7-27.4]	govt hospital - national/ regional referrral	8.7	[7.1-10.7]
				govt hospital - district	10.2	[8.5-12.3]
				religious - secondary	5	[3.8-6.6]

				private- secondary	0.5	[0.3-0.8]
	primary	22.6	[20.3-25.2]	govt - primary (health center, dispensary, clinic)	18.8	[16.6-21.3]
				religious - primary	2.7	[1.9-3.8]
				private - primary	1	[0.7-1.6]
	other	0.1	[0.0-0.3]	other	0.1	[0.0-0.3]

Figure S1. Care-seeking itinerary for newborns' fatal illness in Malawi, Mozambique, and Tanzania.

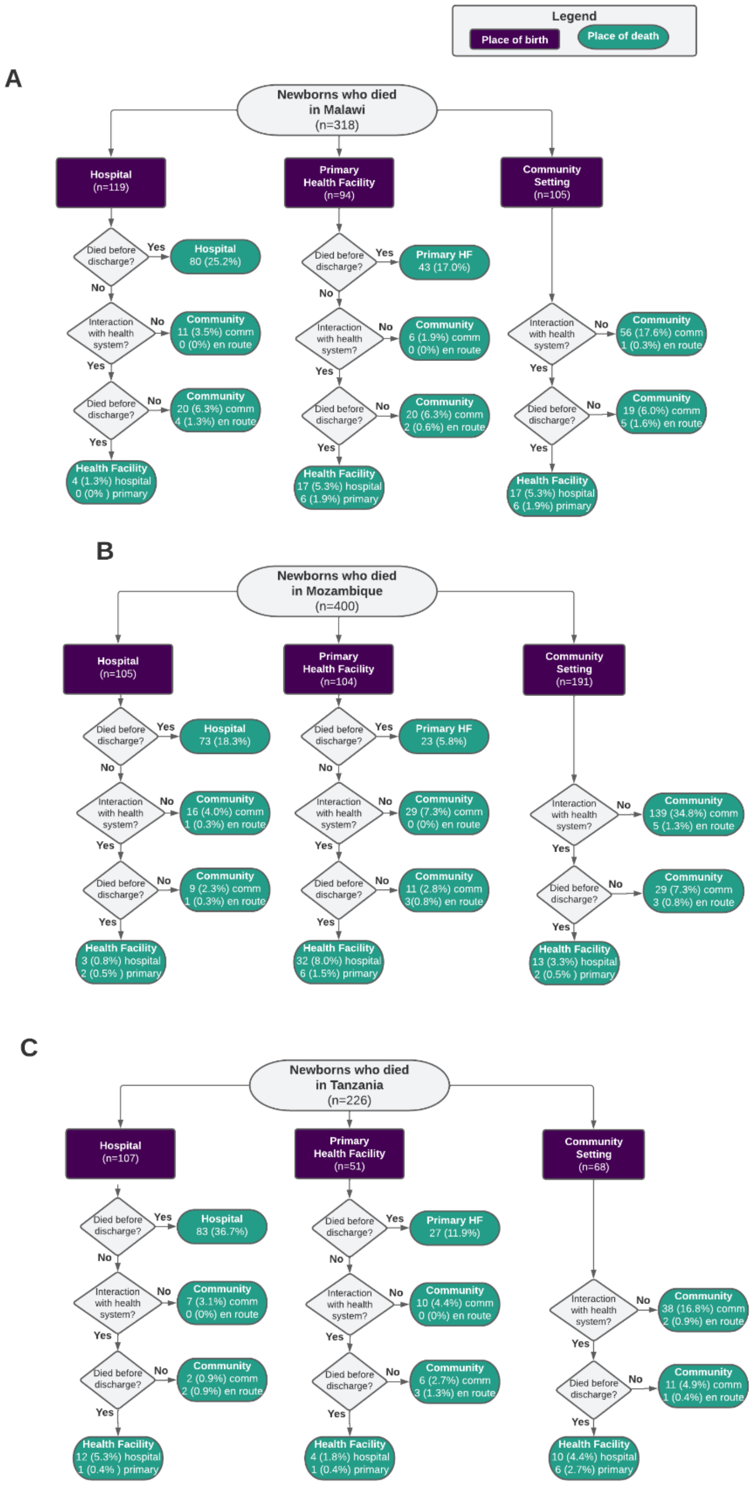

Note: Percentages are calculated by dividing the number of newborns who died in a particular place of birth and place of death category (green ovals) by the total number of newborns who died (318 in Malawi, 400 in Mozambique, and 226 in Tanzania).

Figure S2 Barriers to care-seeking reported by caretakers of newborns who died in Malawi, Mozambique, and Tanzania

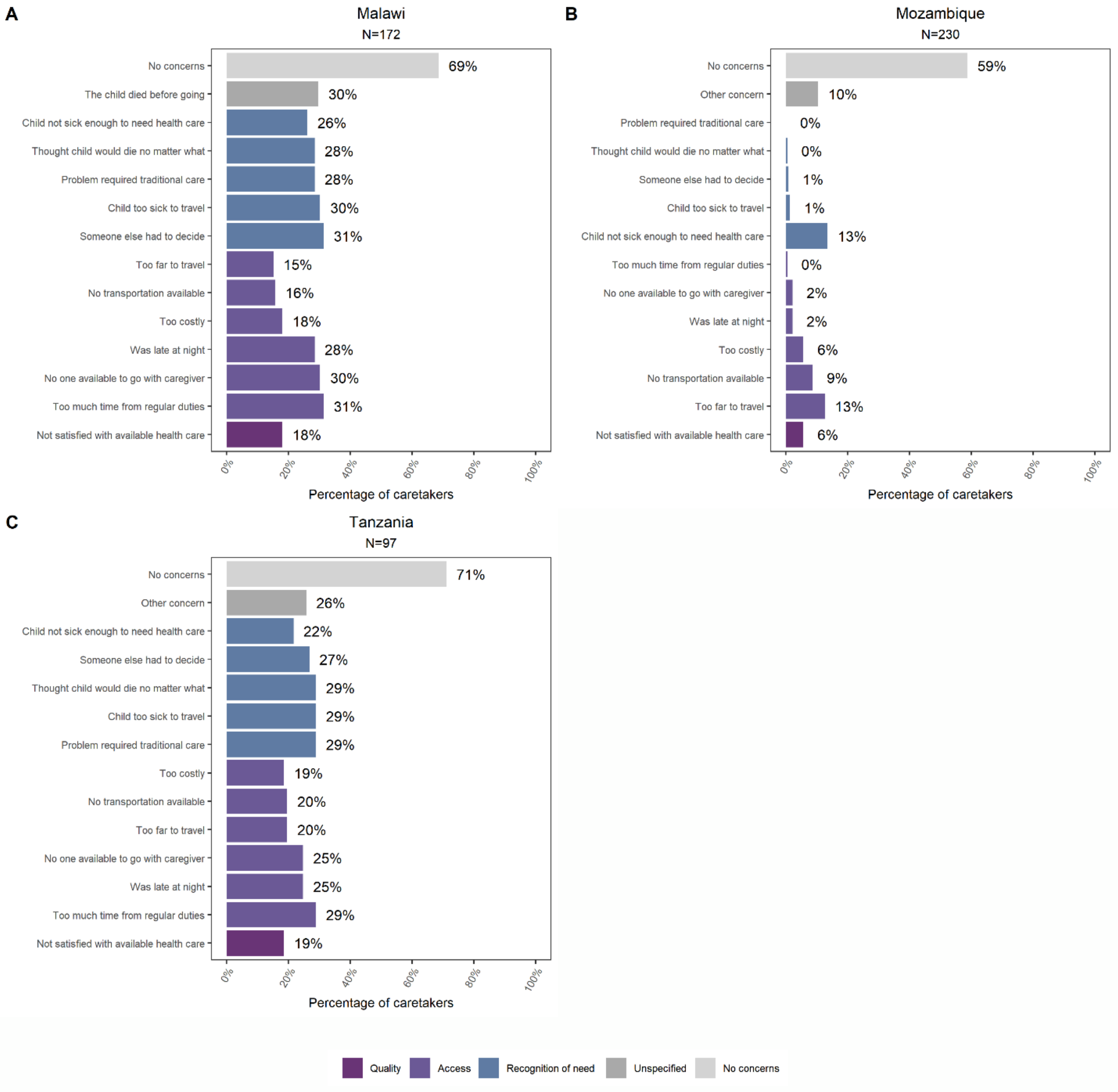

Table S3. Detailed definitions and survey-specific availability for items used to measure facility readiness for each of the eight interventions required for small and sick newborn care included in the analysis. Interventions included are essential newborn care (ENC), breastfeeding, resuscitation, prevention of mother-to-child transmission of HIV (PMTCT), kangaroo mother care (KMC), antibiotics for neonatal infection, and general readiness items (cross-cutting).

Domain	Item	Detailed indicator definition	Data availability notes	Survey specific: Indicator not collected	Cross-country differences
**Essential newborn care (ENC)** – 9 items					
Equipment and supplies	Linen for drying baby	Linen for wrapping and drying the newborn observed available in the delivery service provision area.	Not collected in SARA; collected for some SPAs	MWI_2013 MOZ_2018	
Equipment and supplies	Cord cutting supplies	Sterile scissors/blade to cut cord and cord clamp OR sterile delivery pack observed available in the delivery service provision area.			MWI and TZA were observed available while MOZ was observed available and functional
Equipment and supplies	Infant scale	Infant weighing scale observed available and functional in the delivery service provision area or OPD.			
Equipment and supplies	Thermometer for low-body temperature	Thermometer for low-body temperature observed available and functional in the delivery service provision area.	Not collected in SARA	MOZ_2018	

Medicines and commodities	Vitamin K	Vitamin K observed in pharmacy or anywhere in the facility where medicines are routinely stored; at least one with valid expiration date.	Not collected in SARA; collected for some SPAs	MWI_2013 MOZ_2018	
Medicines and commodities	Antibiotic eye ointment for newborns (tetracycline or other)	Antibiotic eye ointment for newborns observed in pharmacy or anywhere in the facility where medicines are routinely stored; at least one with valid expiration date.			
Medicines and commodities	Chlorhexidine solution[3]	Chlorhexidine solution observed in pharmacy or anywhere in the facility where medicines are routinely stored; at least one with valid expiration date.	Not collected in SARA	MOZ_2018	Not collected in MOZ; CHX not part of national policy for TZA so excluded for TZA
Trained staff	Staff trained in clean cord cutting and appropriate cord care	Proportion of health care workers delivering newborn care services that have been trained in clean cord cutting and appropriate cord care in the last two years.	Not collected in SARA	MOZ_2018	
Trained staff	Staff trained in thermal care	Proportion of health care workers delivering newborn care services that have been trained in thermal care (including immediate drying and skin-to-skin care) in the last two years.	Not collected in SARA	MOZ_2018	
**Breastfeeding** – 2 items					

[3] Only expected in countries for which chlorhexidine is part of the national policy.

Trained staff	Staff trained in early and exclusive breastfeeding	Proportion of health care workers delivering newborn care services that have been trained in early and exclusive breastfeeding in the last two years.	Not collected in SARA	MOZ_2018	
Routine services	Initiation of breastfeeding within the first hour	Facility routinely practices initiation of breastfeeding within the first hour.			
**Resuscitation** – 5 items					
Equipment and supplies	Airway suction apparatus (suction apparatus with catheter or suction bulb for mucus extraction)	Airway suction apparatus (suction apparatus with catheter or suction bulb for mucus extraction) observed available and functional in the delivery service provision area.			
Equipment and supplies	Infant resuscitation bag/mask	Infant resuscitation bag/mask observed available and functional in the delivery service provision area.			
Equipment and supplies	Stethoscope	Stethoscope observed available and functional in the delivery service provision area.			MOZ used item in the OPD as not collected in the delivery service area
Trained staff	Staff trained in neonatal resuscitation using bag and mask	Proportion of health care workers delivering newborn care services that have been trained in neonatal resuscitation using bag and mask in the last two years.			MOZ used alternative definition while SPA countries

		Alternative: At least one health care worker providing delivery services has been trained in neonatal resuscitation using bag and mask in the last two years.			used preferred definition
Routine services	Facility past three months provided neonatal resuscitation	Providers have carried out neonatal resuscitation as part of their work in the facility in the past three months.			MOZ definition is past 12 months
**Prevention of mother-to-child transmission of HIV (PMTCT)** – 8 items					
Infrastructure	PMTCT room is private room with auditory and visual privacy	PMTCT room is private room with auditory and visual privacy.			MWI and TZA have option for PMTCT room within delivery area or as a separate service area; MOZ only asks as a separate service area; SPA countries were observed while MOZ/SARA was reported
Medicines and commodities	Antiretrovirals for babies	Antiretrovirals for babies (NVP or AZT syrup only) observed in pharmacy or anywhere in the facility where medicines are routinely stored; at least one with valid expiration date.			

Medicines and commodities	Antiretrovirals for mothers	Antiretrovirals for mothers (country-specific first line ARV prophylaxis for HIV-positive pregnant women) observed in pharmacy or anywhere in the facility where medicines are routinely stored; at least one with valid expiration date.			TZA: TDF/3TC/EFV or AZT/3TC/NVP MWI: TDF/3TC/EFV OR AZT/3TC/NVP MOZ: TDF/3TC/EFV or AZT/3TC/EFV or TDF/3TC/LPV)
Diagnostics	HIV diagnostic capacity	Facility has the ability to conduct HIV testing onsite and has the required equipment available and functioning/unexpired (RDT, ELISA (scanner/reader, washer, assay, and incubator), dynabeads, or Western blot).			MWI did not ask expiration of western blot or availability of ELISA assay; MOZ did not ask Western blot test or dynabeads
Guidelines	PMTCT guidelines	Guidelines for PMTCT are available in the PMTCT service provision area.			
Guidelines	Infant and young child feeding counseling guidelines	Guidelines for infant and young child feeding are observed available within the PMTCT service provision area.			
Trained staff	Staff trained PMTCT or ARV prophylactic	Proportion of health care workers delivering PMTCT services that have been trained in PMTCT or ARV			MOZ used alternative definition while

	treatment for PMTCT	prophylactic treatment for PMTCT in the last two years.<br>Alternative: At least one health care worker providing PMTCT services has been trained in PMTCT in the last two years.			SPA countries used preferred definition
Trained staff	Staff trained newborn nutrition counseling of mother with HIV or infant and young child feeding	Proportion of health care workers delivering PMTCT services that have been trained in infant and young child feeding in the last two years.<br>Alternative: At least one health care worker providing PMTCT services has been trained in infant and young child feeding in the last two years.			MOZ used alternative definition while SPA countries used preferred definition
**Kangaroo mother care (KMC)** – 3 items					
Infrastructure	Separate room or space for KMC	Facility has a separate room or space for KMC.	Not collected in SARA	MOZ_2018	
Trained staff	Staff trained in KMC for low-birth-weight babies	Proportion of health care workers delivering newborn care services that have been trained in KMC for low-birth-weight babies in the last two years.	Not collected in SARA	MOZ_2018	
Routine services	Facility practices KMC	Facility practices KMC.			MOZ SARA specifies "has practiced in the last 12 months" whereas MWI and TZA SPA more broadly asks "Does the facility

					practice KMC?"
**Antibiotics for neonatal infection** – 3 items					
Equipment and supplies	Medication delivery mechanism (infusion kit + any IV fluid OR single use syringes)	Medication delivery mechanism (infusion kit + any IV fluid OR single use syringes) observed anywhere in the facility where supplies are routinely stored.			
Medicines and commodities	Antibiotic treatment for neonatal infection	Antibiotic treatment for neonatal infection (Outpatient treatment: Gentamycin (IM or IV) and Amoxicillin (syrup/oral suspension) OR Inpatient treatment: (Benzylpenicillin (IV or IM) OR Ampicillin (IV or IM)) AND Gentamycin (IM or IV)) observed in pharmacy or anywhere in the facility where medicines are routinely stored; at least one with valid expiration date.			
Trained staff	Staff trained in newborn infection management (including injectable antibiotics)	Proportion of health care workers delivering newborn care services that have been trained in newborn infection management (including injectable antibiotics) in the last two years.	Not collected in SARA	MOZ_2018	
**General readiness items** – 7 items					
Equipment and supplies	Thermometer	Thermometer observed available and functional in the delivery service provision area or OPD.			MOZ used item in the OPD only as not collected

					in the delivery service area
Equipment and supplies	Pedal bin waste receptacle with lid and plastic liner	Pedal bin waste receptacle with lid and plastic liner observed available in the delivery service provision area.			MOZ used item in the OPD as not collected in the delivery service area
Equipment and supplies	Environmental disinfectant	Disinfectant (e.g., chlorine, hibitane, alcohol) is observed available in the delivery service provision area or OPD.			MOZ used item in the OPD only as not collected in the delivery service area
Equipment and supplies	Clean/sterile gloves	Disposable latex gloves are observed available in the delivery service provision area.			
Equipment and supplies	Sharps container	Sharps container observed available in the delivery service provision area.			MOZ used item in the OPD as not collected in the delivery service area
Equipment and supplies	Soap and water for handwashing	Handwashing soap and running water OR alcohol-based hand rub observed available in the delivery service provision area.			MOZ used item in the OPD as not collected in the delivery service area
Guidelines	Guidelines containing information on	Guidelines containing information on newborn care (e.g., ENC, IMPAC, BEmOC, CEmOC) are observed			TZA: BEmOC, CEmOC, newborn care

	newborn care (e.g., ENC, IMPAC, BEmOC, CEmOC)	available in the delivery service provision area.			guidelines<br>MWI: BEmOC, CEmOC, IMPAC<br>MOZ: ENC, essential childbirth care

Table S4. Average health facility readiness scores for items required for small and sick newborn care, by intervention and country. Scores reflect the mean across all facility types included in each country's most recent HFA.

SSNC Interventions	Item Number	Readiness Item	Readiness Scores		
			Malawi	Mozambique	Tanzania
Essential Newborn Care (ENC)	1	Linen for drying baby	NA	NA	0.12
	2	Cord cutting supplies	0.95	0.95	0.92
	3	Infant scale	0.95	0.95	0.80
	4	Thermometer for low-body temperature	0.08	NA	0.04
	5	Vitamin K	NA	NA	0.03
	6	Antibiotic eye ointment for newborns (Tetracycline or other)	1.00	0.93	0.51
	7	Chlorhexidine solution	0.36	NA	NA
	8	Staff trained in clean cord cutting and appropriate cord care	0.43	NA	0.43
	9	Staff trained in thermal care	0.43	NA	0.42
Breastfeeding	10	Staff trained in early and exclusive breastfeeding	0.36	NA	0.40
	11	Initiation of breastfeeding within the first hour	0.99	0.99	0.98
Resuscitation	12	Airway suction apparatus (suction apparatus with catheter or suction bulb for mucus extraction)	0.91	0.17	0.74
	13	Infant resuscitation bag/mask	0.89	0.76	0.76
	14	Stethoscope	0.78	0.80	0.72
	15	Staff trained in neonatal resuscitation using bag and mask	0.52	0.42	0.51
	16	Facility past three months provided neonatal resuscitation	0.88	0.85	0.52
Prevention of mother-to-child transmission of HIV (PMTCT)	17	PMTCT room is a private room with auditory and visual privacy	0.79	0.64	0.73
	18	HIV diagnostic capacity	0.97	0.99	0.91
	19	Antiretrovirals for babies	0.87	0.79	0.59
	20	Antiretrovirals for mothers	0.97	0.83	0.80
	21	PMTCT guidelines	0.57	0.72	0.70
	22	IYCF counselling guidelines	0.33	0.46	0.32
	23	Staff trained in PMTCT or ARV prophylactic treatment for PMTCT	0.38	0.48	0.36
	24	Staff trained in newborn nutrition counseling of mother with HIV or IYCF	0.31	0.37	0.29
Kangaroo mother care (KMC)	25	Separate room or space for KMC	0.16	NA	0.04
	26	Staff trained in KMC for LBW babies	0.29	NA	0.32
	27	Facility practices KMC	0.56	0.83	0.21
Antibiotics for neonatal infection	28	Antibiotic treatment for neonatal infection	0.89	0.68	0.28
	29	Medication delivery mechanism (infusion kit + any IV fluid OR single use syringes)	0.99	1.00	0.92

	**30**	Staff trained in newborn infection management (including injectable antibiotics)	0.28	NA	0.30
**General readiness items**	**31**	Thermometer	0.84	0.85	0.64
	**32**	Pedal bin waste receptacle with lid and plastic liner	0.47	0.66	0.55
	**33**	Environmental disinfectant	0.84	0.75	0.78
	**34**	Clean/sterile gloves	0.97	0.87	0.86
	**35**	Sharps container	0.97	0.96	0.93
	**36**	Soap and water for handwashing	0.75	0.82	0.67
	**37**	Guidelines containing information on newborn care	0.66	0.58	0.66

Table S5. Average overall SSNC readiness score and readiness-adjusted institutional delivery coverage, by facility type in Malawi, Mozambique, and Tanzania.

Facility Type	Overall SSNC Mean Facility Readiness Score	Readiness-adjusted institutional delivery coverage (95% CI)
**Malawi**		
Home	0	0.086 (0.076-0.081)
Public - hospital	0.73	0.274 (0.256-0.265)
Public - health centre	0.64	0.503 (0.482-0.493)
Public - health post	0.62	0.011 (0.008-0.009)
CHAM - hospital	0.74	0.057 (0.045-0.051)
CHAM - health centre/post	0.62	0.048 (0.039-0.044)
Private - any type	0.60	0.022 (0.016-0.019)
**Mozambique**		
Home	0	0.302 (0.265-0.284)
Public - hospital	0.90	0.225 (0.198-0.212)
Public - primary	0.80	0.469 (0.43-0.45)
Private - any type	0.75	0.004 (0.001-0.003)
**Tanzania**		
Home	0	0.374 (0.347-0.361)
Public - natl hospital	0.67	0.016 (0.013-0.015)
Public - regional hospital	0.73	0.099 (0.085-0.092)
Public - district hospital	0.70	0.126 (0.111-0.118)
Public - health centre	0.61	0.121 (0.107-0.114)
Public - dispensary/clinic	0.51	0.144 (0.129-0.137)
Mission - hospital	0.72	0.071 (0.059-0.065)
Mission - health centre	0.66	0.017 (0.012-0.015)
Mission - dispensary/clinic	0.54	0.007 (0.005-0.006)
Private - hospital	0.63	0.012 (0.009-0.011)
Private - health centre	0.57	0.005 (0.003-0.004)
Private - dispensary	0.43	0.006 (0.003-0.005)
Private - clinic	0.56	0.001 (0-0)
